# Social contact, social support, and cognitive health in a population-based study of middle-aged and older men and women in rural South Africa

**DOI:** 10.1016/j.socscimed.2020.113167

**Published:** 2020-09

**Authors:** Guy Harling, Lindsay C. Kobayashi, Meagan T. Farrell, Ryan G. Wagner, Stephen Tollman, Lisa Berkman

**Affiliations:** a Institute for Global Health. University College London, United Kingdom; b Africa Health Research Institute, KwaZulu-Natal, South Africa; c Harvard Center for Population and Development Studies, Harvard University, Cambridge, MA, USA; d MRC/Wits Rural Public Health and Health Transitions Research Unit (Agincourt), School of Public Health, University of the Witwatersrand, Johannesburg, South Africa; e Department of Epidemiology, School of Public Health, University of Michigan, Ann Arbor, MI, USA; f Umeå Centre for Global Health Research, Umeå University, Umeå, Sweden; g INDEPTH Network, Accra, Ghana; h Department of Social and Behavioral Sciences, Harvard T.H. Chan School of Public Health, Cambridge, MA, USA

**Keywords:** Cognitive function, Cognitive impairment, Social networks, Social support, Educational attainment, Gender, South Africa

## Abstract

**Background:**

Several theories seek to explain how social connections and cognitive function are interconnected in older age. These include that social interaction protects against cognitive decline, that cognitive decline leads to shedding of social connections and that cognitive decline leads to increased instrumental support. We investigated how patterns of social contact, social support and cognitive health in rural South Africa fit with these three theories.

**Method:**

We used data from the baseline of “Health and Aging in Africa: a Longitudinal Study of an INDEPTH community in South Africa” (HAALSI), a population-based study of 5059 individuals aged ≥ 40 years. We evaluated how a range of egocentric social connectedness measures varied by respondents' cognitive function.

**Results:**

We found that respondents with lower cognitive function had smaller, denser social networks that were more local and more kin-based than their peers. Lower cognitive function was associated with receipt of less social support generally, but this difference was stronger for emotional and informational support than for financial and physical support. Impairment was associated with greater differences among those aged 40–59 and those with any (versus no) educational attainment.

**Conclusions:**

The patterns we found suggest that cognitively impaired older adults in this setting rely on their core social networks for support, and that theories relating to social connectedness and cognitive function developed in higher-income and higher-education settings may also apply in lower-resource settings elsewhere.

## Highlights

• Theories of social engagement and cognition were developed in high-income settings.
• We tested the hypotheses among rural South Africans with low educational attainment.
• Respondents with low cognitive function had smaller, denser, kin-based social worlds.
• Differences in instrumental support were less than those for emotional support.
• Patterns seen in higher-income settings were replicated in this poorer African one.

## 1 Introduction

Cognitive impairment is an increasing cause of morbidity in low-and-middle income countries (LMICs) and prevalence at any age is higher than that seen in higher-income settings. This ubiquity has substantial economic and social implications (Skirbekk et al., 2012), although the social drivers of cognitive health in older LMIC populations are not well understood (Lekoubou et al., 2014; Olayinka and Mbuyi, 2014). Cognitive aging is a complex function of biological processes, genetic inheritance, psychological factors, and social interaction (Inui, 2003). Interpersonal interaction is one modifiable factor through which to protect against cognitive decline (Richards et al., 2007; Wilson et al., 2013), but most evidence pertaining to this relationship has been conducted in higher-income settings.

In this study we aimed to evaluate how interpersonal interaction, particularly social contact and social support, is associated with cognitive function in a cohort of older rural South African adults who had limited educational opportunities. We also aimed to determine to what extent these patterns fit with hypothesized associations based on theories proposed in higher-income settings.

Interpersonal interactionbroadly conceived (hereafter "social connection"), is hypothesized to protect against cognitive decline (Berkman et al., 2000). At least three categories of causal processes have been proposed linking social connection and cognitive decline. First, greater social stimulation protects against cognitive decline by engaging cognitive skills and strengthening neural connections, i.e., the “use it or lose it” hypothesis (Hultsch et al., 1999). There exists substantial longitudinal observational and trial evidence that higher levels of social contact and social engagement are associated with less cognitive decline (Barnes et al., 2004; Ertel et al., 2008; Hikichi et al., 2017; Mortimer et al., 2012) and that less social connection is associated with incident cognitive decline and dementia (Bassuk et al., 1999; Kuiper et al., 2015; Penninkilampi et al., 2018). In this literature, several social connection indicators have been examined, including social contact count, frequency of contact with friends or family, levels of emotional support, and attendance at religious or community events.

In addition to less social connection leading to cognitive decline, cognitive decline is also theorized to lead to social disengagement. This latter situation may arise if impaired individuals are less able to engage with others, or others are less able or less motivated to engage with them. Empirical evaluations of impaired cognitive function leading to social disengagement are relatively rare (Aartsen et al., 2002; Hosking et al., 2017; B. J. Small et al., 2012). In practice, the association is likely to be either a virtuous circle, whereby better cognitive health promotes ability to engage in stimulating social relationships and activities that further promote cognitive health, or a vicious circle, in which low social engagement and poor cognitive function negatively impact each other (Bosma et al., 2002; Hultsch et al., 1999). This divergence is likely to be exacerbated by perceived loss of cognitive capacity, which can lead to greater disengagement independent of measured cognitive ability (Farrell et al., 2014).

At the same time, a third social process might be expected, whereby older adults experiencing cognitive decline begin to receive more care-related support. This support is likely to be primarily instrumental, including physical and financial assistance, but may also include emotional support. Social contacts may also shift from providing emotional to instrumental support as they notice cognitive decline.

Importantly, the shedding of social connections and accretion of care receipt that may be associated with cognitive decline is likely to be non-random. Following the Convoy Model (Antonucci and Akiyama, 1995), the closest social connections are most likely to be maintained as cognitive capacity declines, while peripheral ties are shed (Clay et al., 2008). Caregiving may be performed most often by those who are geographically near, and by children and other close kin for whom there is expected intertemporal reciprocity (Schulz and Martire, 2004). These closest social ties are likely to be strongly interconnected. A recent cross-sectional comparison of US individuals with normal cognitive function, mild cognitive impairment, and mild Alzheimer's disease showed a progressive shift towards smaller, denser, and more kin-focused social networks with greater symptomology (Perry et al., 2017).

Existing research on these causal processes between social connection and cognitive decline has largely been conducted in higher-income countries with more-or-less nuclear family structures and relatively high levels of educational attainment. It is not clear how context-specific associations between social connectedness and cognitive health might be. Although norms of caregiving and mutual reciprocity beyond immediate relations are fluid in many LMICs, they often remain stronger than in higher-income settings (Knight et al., 2016; Ugargol and Bailey, 2018).

Education is perhaps the strongest known individual-level protective factor against aging-related cognitive decline and dementia (Clouston et al., 2015; Nguyen et al., 2016). Social connection may be more important in the absence of education, replacing its protective role in providing a cognitive reserve, i.e., acting as a buffer against cognitive loss in the presence of brain pathology (Chapko et al., 2018; Meng & D'arcy, 2012). Alternatively, social connection may be less effective without education, benefiting only those with some minimum level of educational attainment. This potential discrepancy might exist because more-educated individuals' connections are better able to protect against cognitive decline, since these connections tend to be better educated themselves (McPherson et al., 2001). The third possibility is that education does not modify any association between social connection and cognitive outcomes.

Evidence of the interplay between social connection and cognitive impairment in populations with limited educational opportunities is needed to better understand the mechanisms that drive associations between social connection, educational attainment, and cognitive function. In many LMICs, school access and quality are limited, especially for those who are now middle-aged or older (Kenn, 2016). Existing evidence in low-education populations among older Spanish adults educated pre-World War II and in Nigeria has found less social contact or engagement to be associated with cognitive decline and dementia (Ejechi, 2015; Gureje et al., 2011; Zunzunegui et al., 2003). Greater involvement in social groups has also been associated with less decline in cognition among older adults, often with limited educational attainment, in both China and Taiwan (Chiao, 2019; Lam et al., 2019).

Older rural black South Africans are an important population in which to study the relationship between social connection and cognition. Black South Africans were systematically excluded from high-quality educational opportunities during Apartheid (1948–1994), able to access only a minimally-financed “Bantu” education system focused on the production of a labor force (Christie and Collins, 1982). In addition to its unique educational history, rural South Africa is an important environment in which to test associations between social connection and cognition, due to its complex intergenerational dynamics driven by economic and population health factors. Formal employment opportunities were, and often still are, limited to those able to migrate for work (Hall and Posel, 2019), resulting in household structures often lacking working-age generations. Furthermore, HIV has taken a substantial toll in rural South Africa since the 1990s, with adult HIV-seropositivity prevalence often above 30%. HIV mortality has limited the level of familial support available to those with cognitive loss (Mojola et al., 2015), and HIV-related morbidity has increased cognitive decline through HIV-associated neurocognitive disorders (Brew and Chan, 2014). Alternative means of developing, maintaining, and promoting cognitive health, such as through social connections, may therefore be particularly important for the current generation of older black South Africans.

We therefore analyzed baseline data from a rural South African cohort of middle-aged and older adults, to determine whether the patterns of social contact and social support observed in this population were consistent with the three causal processes described above. Specifically, we hypothesized that individuals with cognitive impairment would have: (1) fewer social contacts; (2) more kin-focused, geographically proximate, and densely connected social contacts; and (3) generally lower levels of social support, offset by more instrumental assistance (physical and financial support). We further investigated how age and educational attainment might modify the relationship between social connection and cognitive impairment. Our cross-sectional data source does not allow us to determine the temporality of these associations. Nevertheless, by providing a description of how social factors and cognitive aging outcomes are patterned in this setting, we aimed to generate hypotheses for future longitudinal data.

## 2 Method

### 2.1 Setting and sample

Our data derive from the 2014-15 baseline round of the population-representative “Health and Aging in Africa: A Longitudinal Study of an INDEPTH community in South Africa” (HAALSI) cohort (Gómez-Olivé et al., 2018). The baseline sample consists of a random selection of adults aged 40 and above in the Agincourt Health and Demographic Surveillance System in Mpumalanga province, South Africa (hereafter, “Agincourt”) (Kahn et al., 2012). Trained interviewers conducted in-home study interviews in the local Shangaan language between November 2014 and November 2015. We included all ages of HAALSI respondents in our analysis, in part to allow evaluation of how our hypotheses varied across age strata.

Agincourt is rural and, while improvements have occurred since the end of Apartheid, it still has limited access to basic services. The area comprises 31 villages (27 in HAALSI) spread across ~420 km^2^; public transport is limited to privately run minibus taxis. Cellphone access was ubiquitous at the household, but not individual, level by 2014-15. Local employment in Agincourt is limited (30% of HAALSI participants aged under 60 were employed at interview), with over 50% of men and 35% of women aged 20–60 working away from home (Collinson et al., 2014). The HIV epidemic has had major impacts in terms of mortality (Kabudula et al., 2017) and morbidity; 23% of HAALSI respondents were HIV seropositive and thus aging with HIV (Rosenberg et al., 2017). Formal support (e.g., institutions, health visitors) for older adults with cognitive impairment is almost non-existent. The wide age range of HAALSI means that participants experienced different aspects of Apartheid and post-Apartheid educational and employment policies, allowing examination of how education may moderate the relationship between social connection and cognitive function in this context.

### 2.2 Measurement of key variables

#### 2.2.1 Cognitive health

Cognitive function was assessed in HAALSI using a battery of validated cognitive measures adapted from the Health and Retirement Study. The battery assessed: orientation in time (ability to state the correct date, month, year, and South African president; four items total); episodic memory (immediate and delayed recall of 10 words read out loud; 20 items total); and ability to count forward from one to 20 (one item) and complete a number pattern (2, 4, 6, ?; one item). First, we used confirmatory factor analysis to generate a latent cognitive *Z*-score based on all measures with a mean of 0 and standard deviation (*SD*) of 1 (Kobayashi et al., 2017). Second, we generated a binary variable for cognitive impairment, comprising those who scored ≤1.5 *SD* below the overall mean on the sum of values for time orientation and episodic memory, or who required a proxy respondent and were reported to have “fair” or “poor” memory (Kobayashi et al., 2019).

#### 2.2.2 Social contact and social support

Our measures of social connection were based on egocentric network data. Egocentric networks capture the connections between respondents (“egos”) and their direct social contacts (“alters”), and sometimes also connections between those contacts. To elicit respondents' current core social connections, respondents were asked to “Please tell me the names of [up to] six adults with whom you have been in communication either in person or by phone or internet in the past six months, starting with the person who is most important to you for any reason.” If the respondent was married and living with their spouse, the spouse's name was added to the list of contacts (“alters”) if not otherwise named. For each alter, we requested sociodemographic information (age, sex, kinship, and residential location), as well as frequency of communication (in-person or by phone/text/email) and how frequently the alter provided emotional, informational, physical, and financial support (Harling et al., 2020a). Finally, respondents were asked how frequently they believed each pair of alters communicated with one another.

We calculated a respondent's level of social contact as: (1) the number of contact names provided (i.e., 0–7); and (2) the estimated number of days per month in which an alter had contact with a respondent, summed across all named alters (i.e., a maximum of 30 × 7 = 210 contact-days). For example, an ego reporting four alters, two of whom they see daily and two of whom they talk to weekly would have 2 × 30 + 2 × 4 = 68 contact-days). We measured social support as the number of support-days received per month in the same manner, but specific to each support type. We measured kinship in binary terms, categorizing alters as either kin or non-kin. We measured ego-alter geographical proximity in four categories: living in the same household; the same village; elsewhere in the Agincourt site; or elsewhere in South Africa. Finally, we measured the effective size of each respondent's egocentric network as a respondent's alter count, minus the average number of ties that each alter has to other alters (Burt, 1992); effective size reflects the breadth of independent input sources respondents have available to them.

#### 2.2.3 Covariates

In addition to age (in decades) and gender, we considered two sets of covariates. First, those reflecting early life experiences: country of origin (approximately one-third of the local population migrated as refugees from Mozambique in the 1980s); educational attainment; self-reported literacy; self-rated childhood health; and father's occupation. Second, those reflecting current sociodemographic characteristics: marital status; household size; employment status; and household wealth.

### 2.3 Statistical analysis

We conducted a complete-case analysis. After describing exposures and outcomes, we conducted multivariable regression using linear models for cognitive function *Z*-scores and Poisson models with robust error variance structure for cognitive impairment (yes vs. no). All models were hierarchical, nesting respondents in interviewers, and adjusted for month of interview, since interviewer identity and interview month systematically affected HAALSI baseline social network responses (Harling et al., 2018). We began by assessing the association between binary cognitive impairment and monthly communication event count, first adjusting for age and gender, and then sequentially adding early life variables and current sociodemographic variables. We then repeated the fully adjusted models for each combination of outcome and social contact/support.

As sensitivity analyses, we reran our fully adjusted models adding in separate models multiplicative interaction terms for social contact/support with: (1) respondent age (<60, ≥60); (2) respondent gender; (3) respondent education level (none vs. any); and (4) household size, to evaluate possible effect modification of the social connection-cognition relationship by these factors. Finally, we assessed whether sources of communication and social support differed according to cognitive status by adding an interaction term between level of communication/support and relationship type to the relevant cognitive impairment models.

## 3 Results

A total of 5059 eligible respondents completed HAALSI baseline questionnaires (85.9% response rate), of whom 5019 had a valid response for the cognitive impairment variable and 4927 had a valid latent cognitive *Z*-score. Forty-eight individuals were missing covariate values, two of whom were also missing cognitive impairment status, and nine missing latent cognitive *Z*-scores. Respondents were, by design, approximately equally men and women (Table 1). Three-quarters were unemployed, educational attainment and literacy were limited, and the great majority lived in households of three or more people. Almost one-third were born in Mozambique and almost all were or had previously been married.

**Table 1:** Descriptive statistics by cognitive impairment status.

	All respondents	No impairment	Cognitive impairment
*N*	5059	4603	416
Men 40-49	8.3%	8.6%	3.8%
50-59	12.3%	12.8%	6.3%
60-69	12.7%	13.1%	8.4%
70-79	8.8%	8.7%	10.3%
80+	4.2%	3.7%	10.3%
Women 40-49	9.9%	10.7%	1.0%
50-59	15.5%	16.4%	7.0%
60-69	13.1%	13.4%	10.3%
70-79	8.5%	7.8%	15.9%
80+	6.6%	4.8%	26.7%
Employment status			
Not working	73.5%	72.2%	88.9%
Employed (part or full time)	15.9%	17.1%	3.4%
Homemaker	10.3%	10.6%	7.7%
Household size			
Living alone	10.6%	10.1%	16.3%
Living with one other person	10.6%	10.3%	14.9%
Living with 2–5 others	48.2%	48.6%	43.8%
Living with 6+ others	30.6%	31.1%	25.0%
Household asset level			
Lowest quintile	20.7%	19.6%	32.0%
Second lowest quintile	19.8%	19.4%	24.8%
Middle quintile	19.6%	19.8%	17.1%
Second highest quintile	19.9%	20.2%	17.5%
Highest quintile	20.0%	21.1%	8.7%
Educational attainment			
No formal education	45.6%	42.2%	85.0%
Some primary (1–7 years)	33.9%	36.0%	12.6%
Some secondary or more (8+ years)	20.1%	21.9%	2.4%
Country of origin:			
Mozambique/other not South Africa	30.2%	28.5%	48.0%
Marital status			
Never married	5.7%	5.5%	8.4%
Separated/divorced	12.8%	13.0%	11.8%
Widowed	30.4%	28.2%	54.6%
Currently married	50.9%	53.3%	25.2%
Can vs. cannot read or write	58.3%	62.8%	9.6%
Father's occupation			
Skilled	49.0%	50.3%	36.1%
Unskilled	28.6%	28.5%	29.9%
Other	11.4%	11.4%	11.3%
Don't know	10.8%	9.7%	22.7%
Childhood self-rated health:			
Good/very good (vs. moderate/bad/very bad)	87.6%	88.3%	81.4%

*Note.* Differences between those with and without cognitive impairment statistically significant (*p* < 0.001) for all variables shown based on Kruskall–Wallis tests (Rank-Sum test for ordinal variable). Forty-eight individuals missing at least one covariate: employment status, *n* = 10; education level, *n* = 17; country of origin, *n* = 5; marital status, *n* = 4; literacy, *n* = 3; paternal occupation, *n* = 12; childhood health, *n* = 4.

**Fig. 1:**
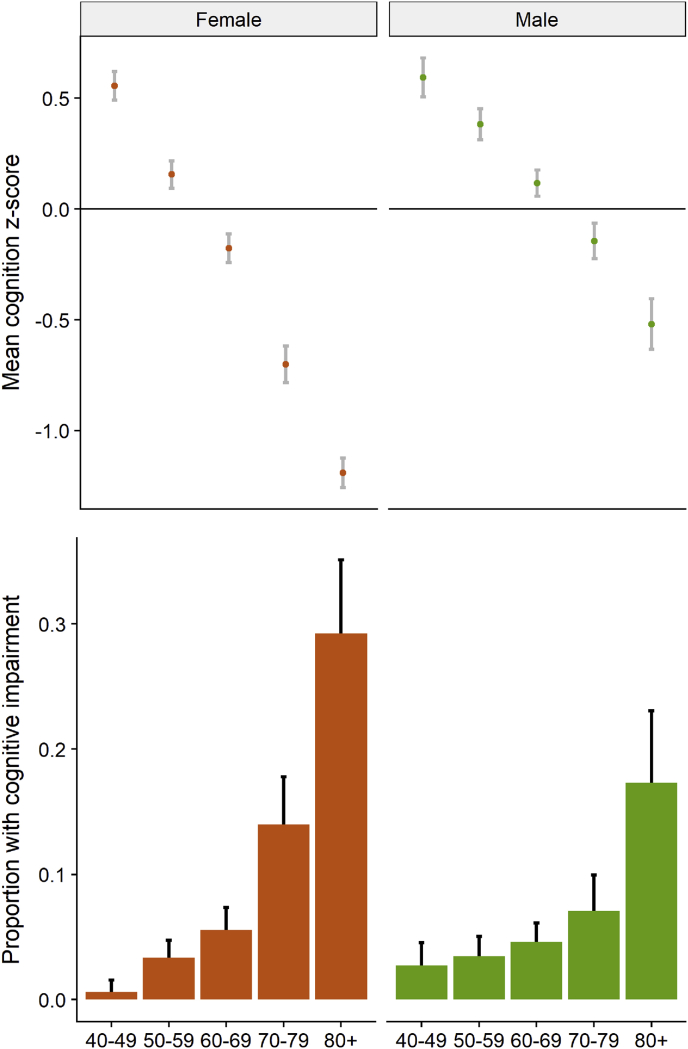
Cognition outcomes in HAALSI baseline by age and gender.

For cognitive impairment, 416 (8.3%) of respondents scored ≤1.5 *SD* below the mean or required a proxy interview with ‘fair’ or ‘poor’ proxy-reported memory. Age was inversely associated with latent cognitive *Z*-score, and positively associated with the likelihood of cognitive impairment; women had worse cognitive function than men, especially after age 70 (Fig. 1). Reflecting this age and gender pattern, cognitive impairment was positively associated with unemployment, living alone, lower household wealth, less education and illiteracy, being a migrant, being widowed, and having worse childhood health status.

On average, respondents named just over three important others and communicated with these alters around 60 times per month (Table 2). Almost four-fifths of contacts were kin, the majority of whom lived either in the same household or village as the respondent. Respondents received informational, emotional, and physical support at roughly the same rate, with financial support being less frequent. Those assessed as cognitively impaired received less contact and less support of all kinds, but had a greater proportion of contacts who were kin. There was little difference in the number of same-household contacts by cognitive status, but those with impairment had notably fewer contacts elsewhere.

**Table 2:** Social contact and support descriptive statistics by cognitive impairment status.

	All respondents		No impairment	Cognitive impairment
	Mean (*SD*) or percent	[IQR]	Mean or percent	Mean or percent
Named contacts	3.1	[2, 4]	3.2	2.0
Communication events per month	60.6 (38.3)	[30, 90]	62.6	40.0
**Contact kinship**				
Named kin	2.4	[1, 3]	2.4	1.7
Any named non-kin	35.5%		37.3%	17.3%
Percent of named contacts who are kin	79.1%	[67%, 100%]	78.5%	86.9%
**Contact distance**				
Same household	0.8	[0, 1]	0.8	0.7
Same village	1.3	[0, 2]	1.3	0.8
Agincourt area	0.4	[0, 0]	0.4	0.2
Elsewhere South Africa	0.5	[0, 1]	0.5	0.2
**Egonet effective size**				
Effective size (range 1–5.7)	1.1	[1, 1]	1.1	1.1
**Support types**				
Informational, per month	29.3 (33.1)	[4, 40]	30.4	17.8
Emotional, per month	26.5 (32.8)	[4, 35]	27.5	16.3
Financial, per month	15.0 (22.2)	[1, 30]	15.5	10.5
Physical, per month	24.5 (26.1)	[4, 34]	25.3	15.5

*Note.* IQR: Interquartile range; *SD*: Standard deviation. *SD* only given when used as a unit of analysis in subsequent regression analysis.

In age and gender-adjusted regressions, a one-*SD* increase in social contact communication per month (38 additional events) was associated with 0.58 (95% confidence interval (CI): 0.48, 0.69) times the risk of having cognitive impairment (Model 1, Table 3). Individuals who reported 22 total communication events per month (one *SD* below the mean) had an adjusted probability of cognitive impairment of 25.6%, compared to 8.5% for those reporting 99 events (one *SD* above the mean). When we added covariates relating to early life circumstances (Model 2), although illiteracy was strongly correlated with greater cognitive impairment, the association of monthly social communication with impairment remained largely unchanged. This pattern was similar when including current sociodemographic characteristics (Model 3): currently being married was associated with lower likelihood of cognitive impairment, but this association had little impact on the communication-impairment association. In the final model, a one-*SD* increase in communication was associated with 0.74 (95%CI: 0.63, 0.87) times lower cognitive impairment.

**Table 3:** Poisson regression models for cognitive impairment and monthly communication event count.

Variable	Model 1		Model 2		Model 3	
Communication events per month (*SD*)	0.58	[0.48, 0.69]	0.64	[0.55, 0.75]	0.71	[0.60, 0.83]
Age and gender (ref: men 40–49)						
Men 50-59	1.08	[0.45, 2.63]	0.93	[0.46, 1.88]	1.14	[0.56, 2.32]
60-69	1.43	[0.73, 2.81]	0.86	[0.49, 1.52]	1.16	[0.73, 1.83]
70-79	2.50	[1.29, 4.85]	1.34	[0.76, 2.35]	1.85	[1.13, 3.05]
80+	4.81	[2.52, 9.15]	2.03	[1.18, 3.48]	2.68	[1.65, 4.35]
Women 40-49			0.22	[0.06, 0.80]	0.28	[0.08, 0.99]
50-59	0.99	[0.40, 2.46]	0.61	[0.28, 1.36]	0.81	[0.39, 1.70]
60-69	1.66	[0.76, 3.63]	0.79	[0.41, 1.54]	0.97	[0.54, 1.76]
70-79	3.58	[1.80, 7.14]	1.39	[0.75, 2.57]	1.64	[0.94, 2.86]
80+	7.24	[3.94, 13.3]	2.39	[1.41, 4.06]	2.83	[1.79, 4.47]
Educational attainment (ref: no formal education)						
Some primary (1–7 years)			0.80	[0.61, 1.05]	0.83	[0.63, 1.10]
Some secondary or more (8+ years)			0.65	[0.33, 1.26]	0.78	[0.41, 1.50]
Can vs. cannot read or write			0.15	[0.11, 0.22]	0.17	[0.12, 0.23]
Country of origin: Mozambique/other vs South Africa			1.17	[0.97, 1.42]	1.20	[1.00, 1.46]
Father's occupation (ref: skilled)						
Unskilled			1.24	[0.98, 1.56]	1.20	[0.97, 1.49]
Other			1.33	[0.96, 1.84]	1.27	[0.93, 1.73]
Don't know			1.57	[1.30, 1.90]	1.59	[1.32, 1.91]
Childhood self-rated health:						
Good/very good vs. moderate/bad/very bad			0.73	[0.57, 0.94]	0.78	[0.61, 1.01]
Marital status (ref: currently married)						
Never married					2.46	[1.76, 3.46]
Separated/divorced					1.48	[1.03, 2.13]
Widowed					1.44	[1.08, 1.91]
Employment status (ref: not working)						
Employed (part or full time)					0.61	[0.36, 1.03]
Homemaker					0.72	[0.46, 1.11]
Household size (ref: living alone)						
Living with one other person					1.25	[0.88, 1.78]
Living with 2–5 others					1.07	[0.76, 1.51]
Living with 6+ others					0.93	[0.72, 1.20]
Household asset level (ref: lowest quintile)						
Second lowest quintile					1.00	[0.79, 1.27]
Middle quintile					0.89	[0.72, 1.09]
Second highest quintile					0.97	[0.73, 1.29]
Highest quintile					0.77	[0.55, 1.07]
Intraclass correlation coefficient		0.203		0.156		0.167

*Note.* Values presented are prevalence rate ratios and [95% confidence intervals]. *N* = 4973 for all models. All models are hierarchical (individuals nested in interviewers) Poisson regressions with robust error variance and contain fixed effects for month of interview. *SD*: Standard deviations.

As with communication events, nearly all social contact and support variables were positively associated with latent cognitive *Z*-scores and negatively associated with cognitive impairment, i.e., better cognition was associated with more social connection (Table 4). Individuals with worse cognitive status named both fewer kin and non-kin contacts, although the difference was more marked for non-kin: those who named non-kin contacts were 55% as likely to be cognitively impaired as those reporting no non-kin contacts. Similarly, respondents with cognitive impairment had fewer contacts living at all distances, but the differences were greatest for those contacts living outside the same household. Each additional beyond-household contact was associated with a 25–30% lower probability of cognitive impairment while each extra within-household contact was associated with only a 15% reduction (Table 4). The effective social network size of those with lower cognitive scores was non-significantly smaller than that of their peers, suggesting that cognitively impaired individuals have a slightly more tightly connected set of social contacts than those without impairment.

**Table 4:** Adjusted regression models for social contact and support and cognitive health.

Variable (unit of change)	Cognitive impairment *		Standardized cognition score (*SD*) **	
	Poisson model		Linear model	
Named contacts (count)	0.75	[0.68, 0.83]	0.03	[0.01, 0.04]
Communication events per month (*SD*)	0.71	[0.60, 0.83]	0.05	[0.02, 0.07]
**Contact kinship**				
Named kin (count)	0.84	[0.77, 0.91]	0.01	[0.00, 0.02]
Any named non-kin (binary)	0.55	[0.42, 0.73]	0.07	[0.03, 0.11]
Percent named contacts who are kin (10 %age points)^†^	1.06	[1.02, 1.09]	−0.01	[-0.02, 0.00]
**Contact distance**^**‡**^				
Same household (count)	0.85	[0.75, 0.97]	−0.02	[-0.05, 0.01]
Same village (count)	0.70	[0.61, 0.80]	0.03	[0.01, 0.05]
Agincourt area (count)	0.75	[0.63, 0.90]	0.06	[0.03, 0.09]
Elsewhere South Africa (count)	0.74	[0.65, 0.86]	0.03	[0.01, 0.05]
**Egonet effective size**				
Effective size (range 1–5.7)^†^	0.87	[0.63, 1.21]	0.04	[0.00, 0.09]
**Support types**				
Informational, per month (*SD*)	0.73	[0.64, 0.82]	0.05	[0.02, 0.07]
Emotional, per month (*SD*)	0.72	[0.63, 0.82]	0.07	[0.05, 0.10]
Financial, per month (*SD*)	0.87	[0.79, 0.96]	0.02	[0.00, 0.05]
Physical, per month (*SD*)	0.78	[0.69, 0.86]	0.03	[0.00, 0.05]

*Note.* Each regression coefficient represents results from a different model, with the exception of ‘contact distance’ models (^‡^), where all four variables were included in a single regression. All models are hierarchical (individuals nested in interviewers) using either Poisson with robust error variance or linear models and are adjusted for age, gender, employment status, household size, household wealth, educational attainment, literacy, marital status, father's occupation, childhood health status, and interview month. ^†^Models for percent of contacts who are kin and egonet effective size are also adjusted for the number of contacts named. *N for ‘cognitive impairment’ models is 4973 and values are prevalence rate ratios (and 95% confidence intervals). **N for ‘cognition score’ models is 4888 and values are the difference in standardized cognition score (in *SD*s) associated with a one-unit higher value of the variables as shown in Table 2. IQR: Interquartile range; *SD*: Standard deviation.

Cognitive status was also negatively associated with receipt of social support. Individuals with poorer cognitive status reported that their social contacts provided all kinds of social support less frequently than their cognitively stronger peers. Each *SD* increase in informational and emotional support was associated with 27% and 28% lower risk of cognitive impairment, respectively; the value for physical support was 22% and just 13% for financial support.

When we considered effect modification by age, we found similar patterns in those aged under and over 60, with some differences (Table 5). The association between overall social contact and cognition was significantly stronger in younger ages: a one-*SD* decrease in communication events was associated with 49% lower probability of cognitive impairment for 40–59 year olds, compared to 23% for older adults. Middle-aged cognitively impaired individuals were even less likely than their older peers to name non-kin contacts. Cognitive impairment also looked different by age in terms of geographical location of contacts: 40–59 year olds with impairment did not have the lower numbers of same-household contacts their elders did, but did have substantially fewer contacts living elsewhere in the Agincourt area. Patterns of social support receipt did not differ much by age, except for financial support where receipt did not differ by impairment among younger respondents. This last finding implies that the smaller number of contacts of middle-aged impaired individuals were providing more intense support.

**Table 5:** Adjusted Poisson regression models for social contact/support and cognitive impairment, including interactions with age or education.

	Respondent age				Respondent education			
Variable	40–59		Age ≥60		None		Any	
Named contacts (count)	0.65	[0.52, 0.79]	0.79	[0.72, 0.86]	0.79	[0.72, 0.86]	0.67	[0.53, 0.83]
Communication events per month (*SD*)	0.50	[0.36, 0.68]	0.79	[0.67, 0.93]	0.75	[0.64, 0.89]	0.63	[0.47, 0.85]
**Contact kinship**								
Named kin (count)	0.79	[0.63, 0.99]	0.86	[0.80, 0.93]	0.85	[0.79, 0.92]	0.83	[0.66, 1.05]
Percent named contacts who are kin (10%age points)^†^	0.46	[0.22, 0.97]	0.61	[0.46, 0.81]	0.65	[0.49, 0.88]	0.25	[0.11, 0.53]
Any named non-kin (binary)	1.05	[0.94, 1.17]	1.06	[1.02, 1.10]	1.04	[1.00, 1.08]	1.20	[1.06, 1.35]
**Contact distance**^**‡**^								
Same household (count)	0.90	[0.60, 1.33]	0.87	[0.77, 0.98]	0.88	[0.78, 0.98]	0.88	[0.63, 1.22]
Same village (count)	0.55	[0.42, 0.73]	0.76	[0.66, 0.87]	0.75	[0.66, 0.84]	0.56	[0.38, 0.83]
Agincourt area (count)	0.44	[0.29, 0.66]	0.83	[0.70, 0.97]	0.78	[0.64, 0.95]	0.68	[0.50, 0.92]
Elsewhere South Africa (count)	0.74	[0.57, 0.95]	0.76	[0.65, 0.89]	0.77	[0.67, 0.88]	0.70	[0.47, 1.05]
**Egonet effective size**								
Effective size (range 1–5.7)^†^	0.97	[0.42, 2.24]	0.84	[0.60, 1.18]	0.84	[0.49, 1.46]	0.90	[0.61, 1.34]
**Support types**								
Information support per month (*SD*)	0.61	[0.36, 1.03]	0.76	[0.68, 0.86]	0.73	[0.65, 0.82]	0.75	[0.56, 1.02]
Emotional support per month (*SD*)	0.62	[0.41, 0.96]	0.75	[0.66, 0.85]	0.74	[0.65, 0.83]	0.70	[0.49, 1.00]
Financial support per month (*SD*)	0.93	[0.69, 1.27]	0.86	[0.78, 0.94]	0.87	[0.79, 0.96]	0.87	[0.65, 1.15]
Physical support per month (*SD*)	0.67	[0.39, 1.16]	0.79	[0.70, 0.88]	0.79	[0.71, 0.87]	0.68	[0.52, 0.90]

*Note.* Each pair of regression coefficients (e.g. 40–59/≥60 on one row) represents results from a different model, except ‘contact distance’ models (^‡^), where all four variables were included in a single regression. All models are hierarchical (individuals nested in interviewers). Poisson regressions with robust error variance and are adjusted for age, gender, employment status, household size, household wealth, educational attainment, literacy, marital status, father's occupation, childhood self-rated health status, and interview month. ^†^Models for kin contact percentage and egonet effective size are also adjusted for the number of contacts named. Values for all models are prevalence rate ratios (and 95% confidence intervals) associated with a one-unit higher value of the variables as shown in the first column. IQR: Interquartile range; *SD*: Standard deviation.

Differences in associations by educational attainment were more limited, although in several respects educated respondents resembled younger respondents. Individuals with schooling had stronger associations than unschooled respondents between cognitive impairment and: lower social contact; less communication; fewer non-household contacts; and naming non-kin as contacts. There were no statistically significant differences in social contact or support by respondent gender and household size (Supplementary Table 1).

Finally, respondents with cognitive impairment obtained a significantly smaller proportion of both overall communication and all specific types of support from spouses and non-relations, compensated for by a larger proportion from other relatives (Supplementary Table 2). Multivariable regression models reflected this pattern, although the differences were not significant in most cases (Supplementary Table 3).

## 4 Discussion

In a rural population of middle-aged and older South Africans, we observed associations between cognitive function and a range of measures of social interaction that are consistent with causal processes seen in other, mostly high-income, settings. The results thus provide support for our *a priori* analytic hypotheses. Individuals with cognitive impairment reported smaller core social networks and less frequent communication with important others (see Table 2). Less social contact may reflect disengagement by others due to the increased difficulty of interaction, or respondents with impairment finding connections increasingly difficult to maintain, or those with more initial connectivity being better able to maintain their cognitive capacity. Furthermore, those with cognitive impairment received less social support of all types, but the difference was greater for informational and emotional support than physical and financial support (see Table 4). This pattern of findings is consistent with a general decline in social connectivity, offset to some extent for financial and physical support, need for which we might expect to be higher for those with cognitive impairment.

Respondents with cognitive impairment had a larger proportion of kin vs. non-kin in their social networks, despite such respondents being substantially less likely to be currently married. Non-spousal kin contact did not vary by cognitive status, even for married individuals, but spousal communication and physical support was lower for cognitively impaired individuals. Non-spousal kin thus appeared to be key for those with cognitive impairment. Stereotypically, such kin are often adult children, but in this setting they may be more varied – especially if working-age children are absent working far away or may have passed away from HIV-related illness. While there is evidence in this setting of important interdependencies between generations (Schatz et al., 2015), limited evidence on the obligations of different kinship relationships in this setting exists – particularly in the context of cognitive decline rather than HIV. Unfortunately, the baseline HAALSI survey did not capture kinship type, so we cannot determine how kin-type varied by cognitive status, but the relational position of the alter (e.g., parent, sibling, offspring, cousin) will be available in wave 2. Examining how kin choose or feel obligated to remain connected to those living with cognitive decline is an important topic for further investigation (Manderson and Block, 2016).

Linked to these kin findings, cognitive impairment was associated with fewer contacts in all geographic locations, although the difference was less for contacts living in the same household than for others. These kin and geographic findings highlight that contact with and support from individuals not bound by reciprocal familial obligations were more infrequent for those with cognitive impairment. Additionally, we found a non-significant association between greater interconnection among contacts and cognitive impairment in the respondent. This association suggests that the absent contacts for respondents with cognitive impairment were more peripheral ones. Together, these results paint a picture of a core support network being retained by those with cognitive impairment.

We find that the patterns described above do not differ substantially by respondent gender or household size, but do find differences by respondent age and education (see Table 5). Specifically, compared to those aged over 60, younger respondents with cognitive impairment have significantly less communication overall, and fewer contacts outside their home but within Agincourt. These findings are consistent with evidence from elsewhere that social engagement is more strongly associated with cognition in mid-life than at older ages (Seeman et al., 2011). They also reflect past evidence that younger members of this cohort report more unmet need for Activities of Daily Living care (Harling et al., 2020b), suggesting that middle-aged adults with cognitive impairment in this population may not have their support needs recognized. Conversely, middle-aged individuals in our sample were able to draw on more same-household support than older adults, potentially reflecting the availability of older as well as younger household members.

Stratifying our sample by age also highlights the importance of identifying social causes of cognitive impairment, in particular by separating those with life-long impairment from those acquiring it in older age, due to dementia, stroke, or otherwise. The distribution of causes of cognitive impairment is likely to look very different at age 45 compared to age 70, and this will lead to different requirements and unmet needs. It is important to also bear in mind that differential associations with age in cross-sectional data may reflect survival bias, i.e., those most supported when younger were more likely to survive into older age to be seen in HAALSI. This possibility is particularly important in the context of the large impact of HIV-related mortality in this area (Kabudula et al., 2017), and the relationship between social support and antiretroviral therapy elsewhere (Nachega et al., 2010).

More-educated HAALSI respondents with cognitive impairment differed more from their non-impaired peers than did less-educated respondents, receiving notably less contact especially from non-household kin. This finding is consistent with evidence from older South Koreans showing that cognitive decline among those with lower education was slower if they were more socially engaged (Lee and Yeung, 2019). We are not able to determine in our data whether social connection benefits the less-educated, or if its absence harms the more-educated, or indeed if more-educated individuals are less able to maintain ties as their cognition declines. One possibility is that, as with middle-aged respondents, cognitive impairment may be less-well recognized in more-educated individuals and thus such people are less well supported. These will be important questions for longitudinal data.

Our results demonstrate that in a setting where formal support from community or government is negligible, those living with cognitive impairment received less social contact and support than their cognitively stronger peers. This outcome is perhaps surprising since this setting has greater expectations of social reciprocity than many of those in which past studies of cognitive impairment and social connection have been conducted. This potential shortfall in support may reflect Agincourt being both human and financial-resource constrained. In resource-constrained situations, demands for social support can be harmful, amounting to overdrawing on available social capital stocks (“resource depletion”), leading to stress and ill-health for all involved (Moskowitz et al., 2013). These demands are particularly intense in the context of the HIV epidemic (J. Small et al., 2017).

Further investigation of all these quantitative patterns in longitudinal data are warranted, including the use of methods that can identify causal direction and evaluate the multiple potential mechanisms responsible for the associations shown. Our baseline findings add to existing evidence of a positive association of social connection and cognition in populations with limited educational attainment (Ejechi, 2015; Gureje et al., 2011; Zunzunegui et al., 2003). This growing literature suggests that there may be a benefit to social connectivity for older adults in LMICs in terms of protecting cognition, and possibly an impact of declining cognition on social connection. Additional qualitative work using observation and narrative data on social interactions between older adults and their social connections would add insight into why social contact changes with cognitive decline, as well as how interaction content differs for social contacts living nearby and further away. Given evidence from elsewhere on the potential for spouses and friends to both positively and negatively affect elder health (Antonucci et al., 2001), such work could provide a deeper understanding of how the qualities of spousal, other kin, and non-kin relationships affect the wellbeing of support provided.

Should the relationships reported in this study prove robust, LMIC policymakers might wish to support the maintenance or even generation of meaningful social connections for those facing cognitive decline. Interventions could be considered at the individual, interpersonal, and community levels. Maintaining existing social contacts might be supported through structured community settings at which older adults could meet, including through churches (over 80% of HAALSI participants give a religious affiliation (Moore et al., 2018)). Such structures might be especially important for those who have recently lost connections, e.g., due to loss of employment or spouse, or who are living alone or have few kin. Continuing engagement and support for those with cognitive decline is likely to require more focus on buffering the necessary effort of social contacts. Such assistance could build on existing programs, including the provision of structured community-organized daycare facilities (there is already one such local facility), in-home care provision by government-employed community health workers, or cash transfers to caregivers mirroring existing unconditional childcare and old-age pension grants. The difficulty of generating effective social connections *de novo* (Glass et al., 2004) suggests that a focus on generating social infrastructure on which social connections can grow organically may be the most efficient approach (Klinenberg, 2015).

### 4.1 Limitations

The most important limitation of this work is the cross-sectional nature of the data analyzed. Given the high likelihood of bi-directional causality between the key variables, we cannot determine in these data whether impairment causes reduced social contact and support, or vice versa. Despite this constraint we have provided evidence consistent with existing theories and longitudinal evidence demonstrating that cognitive impairment leads to changes in social connectivity, and that changes in social connectivity lead to cognitive impairment. Our data on social contact and support were self-reported, and thus potentially susceptible to social desirability and recall bias. If these self-reports were differential by cognitive function or impairment, our associational measures may be biased. It is possible that measurement error may be more common among those with cognitive impairment, due to fatigue or recall failure; while this may temper our interpretation of the overall lower level of social connection in amongst those with impairment, it is more difficult to see how this could have led to the differential associations seen by kin, geography, and support type. Despite these limitations, our data are some of the first to explore the associations between social connection and cognitive decline in low income settings. Our analyses are likely generalizable to similar rural settings in South Africa, notably previously designated Black South African areas that remain socioeconomically deprived (Seekings, 2011), but often with diverging inter-household trajectories (Neves, 2017). Replication will be needed in other LMIC settings before determining broader generalizability.

## 5 Conclusions

In this cross-sectional analysis of over 5000 middle-aged and older adults in rural South Africa, we observed that respondents with cognitive impairment had smaller, denser social networks that were more local and kin-based than their peers. These cognitively impaired individuals received less social support in general, but instrumental support was somewhat maintained. These patterns suggest that cognitively impaired older adults in this setting rely on their core social networks for support and that theories developed in higher-income and higher-education settings regarding social connection and cognitive aging may also apply here.

## Declaration of competing interest

None.

## Data Availability

All data underlying this study are publicly available at https://doi.org/10.7910/DVN/F5YHML.
