# Supplementary material for: Social contact, social support, and cognitive health in a population-based study of middle-aged and older men and women in rural South Africa
Source: Soc Sci Med. 2020 Sep;260:113167. doi: 10.1016/j.socscimed.2020.113167 (PMC7441312; doi:10.1016/j.socscimed.2020.113167)
Supplement: Multimedia component 2 [file mmc2.docx]

**Title:** Social contact, social support and cognitive health in a population-based study of middle-aged and older men and women in rural South Africa

# Supplementary Materials

Supplementary Table 1. Multivariable regression models for social contact/support variables & cognitive impairment including interaction terms for respondent characteristics

|  |  | **Respondent age** | | | | | | | |  | **Respondent education** | | | | | | | |
| --- | --- | --- | --- | --- | --- | --- | --- | --- | --- | --- | --- | --- | --- | --- | --- | --- | --- | --- |
|  |  | **Age 40-59** | |  | **Age ≥60** | |  | ***χ^2^*** | **p** |  | **None** | |  | **Any** | |  | ***χ^2^*** | **p** |
|  | Named contacts (count) | 0.65 | [0.52, 0.79] |  | 0.79 | [0.72, 0.86] |  | 4.79 | 0.03 |  | 0.79 | [0.72, 0.86] |  | 0.67 | [0.53, 0.83] |  | 2.03 | 0.15 |
|  | Communication events per month (SD) | 0.50 | [0.36, 0.68] |  | 0.79 | [0.67, 0.93] |  | 8.41 | 0.00 |  | 0.75 | [0.64, 0.89] |  | 0.63 | [0.47, 0.85] |  | 1.28 | 0.26 |
| **Contact kinship** | |  |  |  |  |  |  |  |  |  |  |  |  |  |  |  |  |  |
|  | Named kin (count) | 0.79 | [0.63, 0.99] |  | 0.86 | [0.80, 0.93] |  | 0.62 | 0.43 |  | 0.85 | [0.79, 0.92] |  | 0.83 | [0.66, 1.05] |  | 0.04 | 0.83 |
|  | Percent named contacts who are kin   (10%age points) ^†^ | 0.46 | [0.22, 0.97] |  | 0.61 | [0.46, 0.81] |  | 0.48 | 0.49 |  | 0.65 | [0.49, 0.88] |  | 0.25 | [0.11, 0.53] |  | 6.83 | 0.01 |
|  | Any named non-kin (binary) | 1.05 | [0.94, 1.17] |  | 1.06 | [1.02, 1.10] |  | 0.00 | 0.96 |  | 1.04 | [1.00, 1.08] |  | 1.20 | [1.06, 1.35] |  | 5.24 | 0.02 |
| **Contact distance ^‡^** | |  |  |  |  |  |  |  |  |  |  |  |  |  |  |  |  |  |
|  | Same household (count) | 0.90 | [0.60, 1.33] |  | 0.87 | [0.77, 0.98] |  | 0.03 | 0.87 |  | 0.88 | [0.78, 0.98] |  | 0.88 | [0.63, 1.22] |  | 0.00 | 1.00 |
|  | Same village (count) | 0.55 | [0.42, 0.73] |  | 0.76 | [0.66, 0.87] |  | 5.00 | 0.03 |  | 0.75 | [0.66, 0.84] |  | 0.56 | [0.38, 0.83] |  | 2.02 | 0.15 |
|  | Agincourt area (count) | 0.44 | [0.29, 0.66] |  | 0.83 | [0.70, 0.97] |  | 10.3 | 0.00 |  | 0.78 | [0.64, 0.95] |  | 0.68 | [0.50, 0.92] |  | 0.50 | 0.48 |
|  | Elsewhere South Africa (count) | 0.74 | [0.57, 0.95] |  | 0.76 | [0.65, 0.89] |  | 0.05 | 0.83 |  | 0.77 | [0.67, 0.88] |  | 0.70 | [0.47, 1.05] |  | 0.20 | 0.66 |
| **Egonet effective size** | |  |  |  |  |  |  |  |  |  |  |  |  |  |  |  |  |  |
|  | Effective size (range 1-5.7) ^†^ | 0.97 | [0.42, 2.24] |  | 0.84 | [0.60, 1.18] |  | 0.10 | 0.75 |  | 0.84 | [0.49, 1.46] |  | 0.90 | [0.61, 1.34] |  | 0.04 | 0.84 |
| **Support types** | |  |  |  |  |  |  |  |  |  |  |  |  |  |  |  |  |  |
|  | Information support per month (SD) | 0.61 | [0.36, 1.03] |  | 0.76 | [0.68, 0.86] |  | 0.70 | 0.40 |  | 0.73 | [0.65, 0.82] |  | 0.75 | [0.56, 1.02] |  | 0.05 | 0.83 |
|  | Emotional support per month (SD) | 0.62 | [0.41, 0.96] |  | 0.75 | [0.66, 0.85] |  | 0.66 | 0.42 |  | 0.74 | [0.65, 0.83] |  | 0.70 | [0.49, 1.00] |  | 0.07 | 0.79 |
|  | Financial support per month (SD) | 0.93 | [0.69, 1.27] |  | 0.86 | [0.78, 0.94] |  | 0.26 | 0.61 |  | 0.87 | [0.79, 0.96] |  | 0.87 | [0.65, 1.15] |  | 0.00 | 0.98 |
|  | Physical support per month (SD) | 0.67 | [0.39, 1.16] |  | 0.79 | [0.70, 0.88] |  | 0.28 | 0.60 |  | 0.79 | [0.71, 0.87] |  | 0.68 | [0.52, 0.90] |  | 1.01 | 0.31 |
|  | Named contacts (count) | 0.74 | [0.65, 0.84] |  | 0.79 | [0.72, 0.88] |  | 1.16 | 0.28 |  | 0.77 | [0.68, 0.87] |  | 0.77 | [0.70, 0.85] |  | 0.00 | 0.99 |
|  | Communication events per month (SD) | 0.72 | [0.56, 0.93] |  | 0.76 | [0.64, 0.90] |  | 0.12 | 0.73 |  | 0.70 | [0.57, 0.87] |  | 0.76 | [0.64, 0.90] |  | 0.52 | 0.47 |
| **Contact kinship** | |  |  |  |  |  |  |  |  |  |  |  |  |  |  |  |  |  |
|  | Named kin (count) | 0.86 | [0.77, 0.95] |  | 0.85 | [0.77, 0.94] |  | 0.02 | 0.87 |  | 0.85 | [0.74, 0.97] |  | 0.86 | [0.79, 0.93] |  | 0.02 | 0.88 |
|  | Percent named contacts who are kin   (10%age points) ^†^ | 1.07 | [1.00, 1.14] |  | 1.04 | [1.01, 1.08] |  | 0.45 | 0.50 |  | 1.07 | [0.99, 1.15] |  | 1.05 | [1.01, 1.09] |  | 0.12 | 0.73 |
|  | Any named non-kin (binary) | 0.48 | [0.29, 0.81] |  | 0.66 | [0.54, 0.79] |  | 1.78 | 0.18 |  | 0.59 | [0.35, 1.02] |  | 0.57 | [0.41, 0.79] |  | 0.01 | 0.90 |
| **Contact distance ^‡^** | |  |  |  |  |  |  |  |  |  |  |  |  |  |  |  |  |  |
|  | Same household (count) | 1.00 | [1.00, 1.00] |  | 1.00 | [1.00, 1.00] |  | 0.84 | 0.36 |  | 0.86 | [0.70, 1.06] |  | 0.89 | [0.78, 1.01] |  | 0.07 | 0.79 |
|  | Same village (count) | 0.80 | [0.58, 1.09] |  | 0.93 | [0.83, 1.05] |  | 0.13 | 0.72 |  | 0.74 | [0.63, 0.88] |  | 0.71 | [0.63, 0.81] |  | 0.29 | 0.59 |
|  | Agincourt area (count) | 0.71 | [0.60, 0.84] |  | 0.73 | [0.64, 0.84] |  | 0.88 | 0.35 |  | 0.82 | [0.64, 1.04] |  | 0.75 | [0.61, 0.93] |  | 0.26 | 0.61 |
|  | Elsewhere South Africa (count) | 0.84 | [0.65, 1.08] |  | 0.73 | [0.61, 0.89] |  | 0.10 | 0.75 |  | 0.73 | [0.60, 0.89] |  | 0.78 | [0.67, 0.89] |  | 0.58 | 0.45 |
| **Egonet effective size** | |  |  |  |  |  |  |  |  |  |  |  |  |  |  |  |  |  |
|  | Effective size (range 1-5.7) ^†^ | 0.83 | [0.48, 1.44] |  | 0.91 | [0.62, 1.34] |  | 0.07 | 0.80 |  | 1.02 | [0.61, 1.71] |  | 0.78 | [0.49, 1.22] |  | 0.61 | 0.44 |

|  |  | **Respondent gender** | | | | | | | |  | **Household size** | | | | | | | |
| --- | --- | --- | --- | --- | --- | --- | --- | --- | --- | --- | --- | --- | --- | --- | --- | --- | --- | --- |
|  |  | **Men** | |  | **Women** | |  | ***χ^2^*** | **p** |  | **1-2 members** | |  | **≥ 3 members** | |  | ***χ^2^*** | **p** |
| **Support types** | |  |  |  |  |  |  |  |  |  |  |  |  |  |  |  |  |  |
|  | Information support per month (SD) | 0.73 | [0.55, 0.98] |  | 0.74 | [0.67, 0.82] |  | 0.01 | 0.93 |  | 0.77 | [0.62, 0.96] |  | 0.73 | [0.64, 0.83] |  | 0.27 | 0.60 |
|  | Emotional support per month (SD) | 0.74 | [0.59, 0.91] |  | 0.73 | [0.65, 0.82] |  | 0.01 | 0.92 |  | 0.74 | [0.62, 0.89] |  | 0.73 | [0.64, 0.83] |  | 0.03 | 0.86 |
|  | Financial support per month (SD) | 0.85 | [0.71, 1.01] |  | 0.89 | [0.81, 0.98] |  | 0.31 | 0.58 |  | 0.87 | [0.70, 1.08] |  | 0.87 | [0.80, 0.96] |  | 0.00 | 0.94 |
|  | Physical support per month (SD) | 0.75 | [0.59, 0.95] |  | 0.78 | [0.67, 0.91] |  | 0.07 | 0.78 |  | 0.77 | [0.65, 0.92] |  | 0.77 | [0.69, 0.86] |  | 0.01 | 0.94 |

Each pair of regression coefficients (e.g. men/women on one row) represents results from a different model, with the exception of ‘contact distance’ models (^‡^), where all four variables were included in a single regression. All models are hierarchical (individuals nested in interviewers) and are adjusted for age, gender, employment status, household size, household wealth, educational attainment, literacy, marital status, father’s occupation, childhood health status and interview month. ^†^Models for percent of contacts who are kin and egonet effective size are also adjusted for the number of contacts named. N for all models is 4973

and values are prevalence rate ratios (and 95% confidence intervals) associated with a one-unit higher value of the variables as shown in the first column. χ^2^ tests test the difference between each pair of regression coefficients (i.e., significance of interaction) and have one degree of freedom. SD: Standard deviation.

Supplementary Table 2. Relationship type composition of provision of monthly social engagement, stratified by cognitive impairment status

|  |  | **N** |  | **Spouse** | |  | **Other relation** | |  | **Non-relation** | |
| --- | --- | --- | --- | --- | --- | --- | --- | --- | --- | --- | --- |
|  |  |  |  |  |  |  |  |  |  |  |  |
| **Communication events** | |  |  |  |  |  |  |  |  |  |  |
|  | Normal cognition | 4,437 |  | 25.1% | [24.2%, 26.0%] |  | 54.0% | [52.9%, 55.2%] |  | 20.8% | [19.9%, 21.8%] |
|  | Cognitively impaired | 320 |  | 13.4% | [10.5%, 16.3%] |  | 73.5% | [69.5%, 77.5%] |  | 13.1% | [10.0%, 16.2%] |
|  | *Z-score* |  |  | *8.0* |  |  | *-9.1* |  |  | *5.3* |  |
| **Information support** | |  |  |  |  |  |  |  |  |  |  |
|  | Normal cognition | 4,241 |  | 29.0% | [28.0%, 30.1%] |  | 52.3% | [51.0%, 53.5%] |  | 18.7% | [17.8%, 19.7%] |
|  | Cognitively impaired | 293 |  | 16.4% | [12.9%, 19.9%] |  | 72.0% | [67.5%, 76.4%] |  | 11.6% | [8.4%, 14.8%] |
|  | *Z-score* |  |  | *7.2* |  |  | *-8.4* |  |  | *5.1* |  |
| **Emotional support** | |  |  |  |  |  |  |  |  |  |  |
|  | Normal cognition | 4,159 |  | 27.1% | [26.1%, 28.1%] |  | 52.6% | [51.4%, 53.9%] |  | 20.3% | [19.3%, 21.2%] |
|  | Cognitively impaired | 281 |  | 16.5% | [12.9%, 20.1%] |  | 70.0% | [65.4%, 74.6%] |  | 13.5% | [10.1%, 16.9%] |
|  | *Z-score* |  |  | *6.6* |  |  | *-7.3* |  |  | *4.3* |  |
| **Financial support** | |  |  |  |  |  |  |  |  |  |  |
|  | Normal cognition | 4,136 |  | 34.9% | [33.7%, 36.1%] |  | 47.5% | [46.2%, 48.8%] |  | 17.6% | [16.6%, 18.5%] |
|  | Cognitively impaired | 282 |  | 18.0% | [14.1%, 22.0%] |  | 71.0% | [66.2%, 75.7%] |  | 11.0% | [7.7%, 14.3%] |
|  | *Z-score* |  |  | *7.6* |  |  | *-8.9* |  |  | *4.8* |  |
| **Physical support** | |  |  |  |  |  |  |  |  |  |  |
|  | Normal cognition | 3,717 |  | 38.4% | [37.1%, 39.8%] |  | 45.8% | [44.4%, 47.2%] |  | 15.8% | [14.8%, 16.8%] |
|  | Cognitively impaired | 254 |  | 19.0% | [14.7%, 23.2%] |  | 69.6% | [64.5%, 74.7%] |  | 11.5% | [8.0%, 15.0%] |
|  | *Z-score* |  |  | *7.4* |  |  | *-8.0* |  |  | *3.5* |  |

Each row sums to 100%. Z-score is the result of a Wilcoxon rank-sum test for a difference in value between the two proportions above each score. All values have p-values <0.001. N for communication/type of support varies due to exclusion of respondents receiving none of the relevant type of social engagement.

Supplementary Table 3. Multivariable regression models for social engagement variables & cognitive impairment
including interaction terms for relationship type of contact

|  |  | **Spouse** | |  | **Other relation** | |  | **Non-relation** | |  | **Spouse vs  other relation** | |  | **Non-relation vs  other relation** | |
| --- | --- | --- | --- | --- | --- | --- | --- | --- | --- | --- | --- | --- | --- | --- | --- |
|  | |  |  |  |  |  |  |  |  |  | ***χ^2^*** | **p** |  | ***χ^2^*** | **p** |
|  | Communication events per month | 0.70 | [0.57, 0.87] |  | 0.84 | [0.74, 0.95] |  | 0.70 | [0.58, 0.85] |  | 1.63 | 0.20 |  | 4.00 | 0.05 |
|  | Information support per month | 0.80 | [0.65, 0.99] |  | 0.85 | [0.77, 0.95] |  | 0.78 | [0.66, 0.91] |  | 0.24 | 0.63 |  | 0.70 | 0.40 |
|  | Emotional support per month | 0.91 | [0.74, 1.10] |  | 0.83 | [0.74, 0.92] |  | 0.77 | [0.66, 0.91] |  | 0.44 | 0.51 |  | 0.48 | 0.49 |
|  | Financial support per month | 0.82 | [0.68, 1.00] |  | 0.97 | [0.89, 1.05] |  | 0.89 | [0.81, 0.98] |  | 1.69 | 0.19 |  | 1.40 | 0.24 |
|  | Physical support per month | 0.77 | [0.66, 0.89] |  | 0.89 | [0.82, 0.97] |  | 0.78 | [0.66, 0.92] |  | 2.40 | 0.12 |  | 1.83 | 0.18 |

Each row represents results from a different regression model. All models are hierarchical (individuals nested in interviewers) and adjust for interview month; multivariable models additionally are adjusted for age, gender, employment status, household size, household wealth, educational attainment, literacy, marital status, father’s occupation and childhood health status. N for all models is 4973 and values are prevalence rate ratios (and 95% confidence intervals) associated with a one-standard deviation higher value of the variables shown in the first column.
